# [Corrigendum] Downregulation of DEC1 inhibits proliferation, migration and invasion, and induces apoptosis in ovarian cancer cells via regulation of Wnt/β-catenin signaling pathway

**DOI:** 10.3892/etm.2025.13020

**Published:** 2025-11-19

**Authors:** Yun Yi, Bing Liao, Ziwen Zheng, Xiaorong Yang, Yunsheng Yang, Yanfang Zhou, Buzhen Tan, Xinfeng Yang

Exp Ther Med 21:372, 2021; DOI: 10.3892/etm.2021.9803

Following the publication of the above article, an interested reader drew to the Editor’s attention that the data panels showing the results of the ‘Invasion/Control’ and Invasion/NC shRNA’ experiments in Fig. 5D on p. 8 contained an overlapping section, such that data which were intended to show the results from differently performed experiments had apparently been derived from the same original source.

Upon re-examining their original data, the authors realized that the data shown in Fig. 5D had been inadvertently assembled incorrectly. The revised version of Fig. 5, now showing data for Fig. 5D from one of the alternative experiments, is shown on the next page. Note that the error made in assembling this figure did not have an impact on either the results or the conclusions reported in the paper. All the authors agree with the publication of this corrigendum, and are grateful to the Editor of *Experimental and Therapeutic Medicine* for allowing them the opportunity to publish this; furthermore, they apologize to the readership for any inconvenience caused.

## Figures and Tables

**Figure 5:**
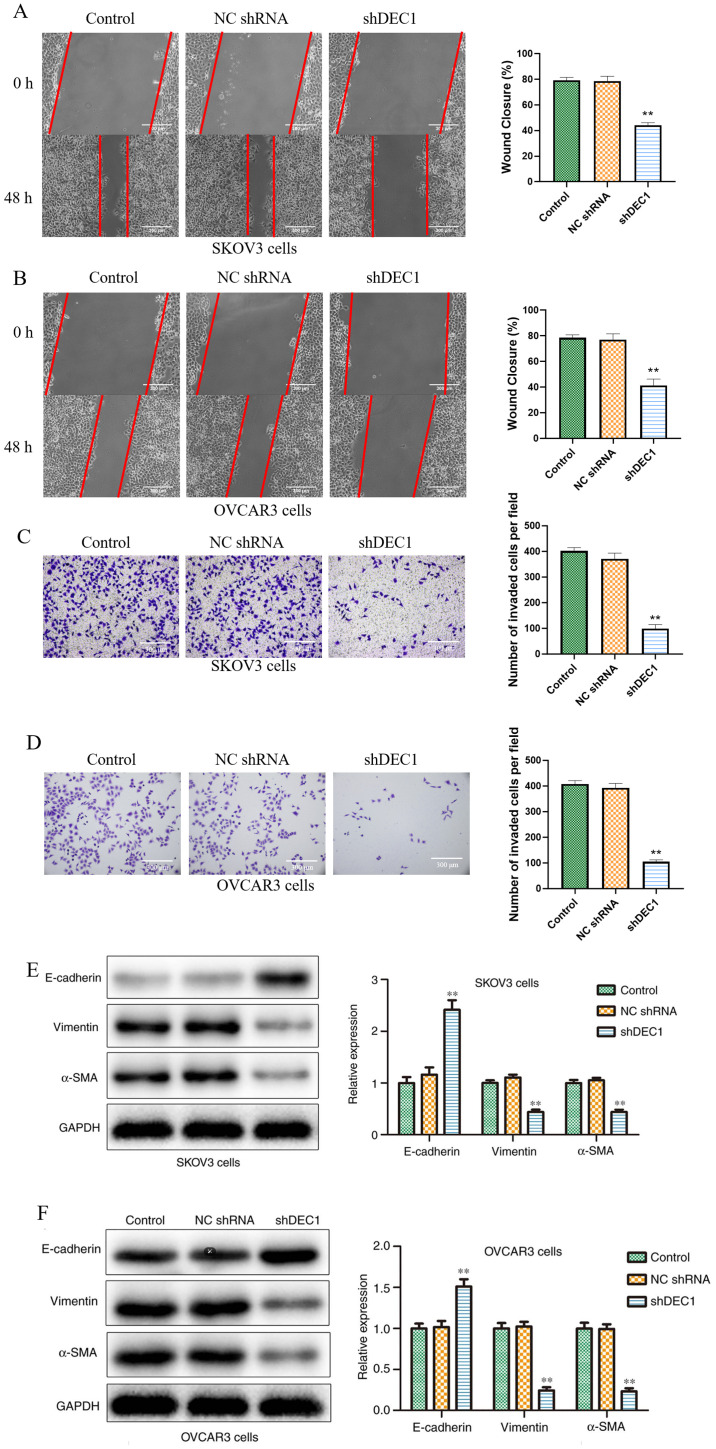
Knockdown of DEC1 inhibits migration and invasion of ovarian cancer cells. Wound healing assay was performed to evaluate the effects of DEC1 on migration in (A) SKOV3 and (B) OVCAR3 cells. Scale bar, 300 µm. Transwell invasion assay was performed to evaluate the effects of DEC1 on invasion in (C) SKOV3 and (D) OVCAR3 cells. Scale bars, 300 µm. Western blotting was conducted to evaluate the effects of DEC1 on the epithelial-mesenchymal transition-related proteins in (E) SKOV3 and (F) OVCAR3 cells. ^**^P<0.01 vs. control (NC) group. NC, negative control; shRNA, short hairpin RNA; DEC1, differential embryo-chondrocyte expressed gene 1; α-SMA, α-smooth muscle actin.

